# What’s New in Prevention of Invasive Fungal Diseases during Hospital Construction and Renovation Work: An Overview

**DOI:** 10.3390/jof9020151

**Published:** 2023-01-23

**Authors:** Ivana Mareković

**Affiliations:** 1 School of Medicine, University of Zagreb, 10 000 Zagreb, Croatia; imarekov@kbc-zagreb.hr; Tel.: +385-1-2367-315; 2 Clinical Department of Clinical Microbiology, Infection and Prevention Control, University Hospital Centre Zagreb, 10 000 Zagreb, Croatia

**Keywords:** *Aspergillus fumigatus*, construction, renovation, healthcare-associated infections, prevention, invasive fungal diseases

## Abstract

The goal of the overview was to give insight into the recent data of invasive fungal diseases (IFDs) associated with construction and renovation in healthcare settings as well as the recent evidence about available prevention and infection control measures. The number of studies describing IFD outbreaks associated with construction or renovation is on the rise again. Applying adequate prevention measures is still a challenge not just for healthcare workers but also for architects and construction workers as well. The role of multidisciplinary teams in the planning and monitoring of prevention measures cannot be overemphasized. Dust control is an inevitable part of every prevention plan. HEPA filters are helpful in the prevention of fungal outbreaks in hematologic patients, but further studies are needed to clarify the extent in which they contribute as specific control measures. The cut-off value for a “threating” level of fungal spore contamination still remains to be defined. The value of antifungal prophylaxis is difficult to assess because other preventive measures are simultaneously applied. Recommendations are still based on few meta-analyses, a large number of descriptive reports, and the opinion of respective authorities. Outbreak reports in the literature are a valuable resource and should be used for education as well as for preparing outbreak investigations.

## 1. Introduction

Invasive fungal diseases (IFDs) are becoming more prevalent worldwide and are responsible for significant morbidity and mortality. Recent studies estimate that fungal infections cause the death of more than 1.5 million people worldwide each year. This is due to the expanding number of patients at risk of these infections, including transplant recipients, cancer patients, patients receiving immunomodulators (e.g., tumor necrosis factor-alpha inhibitors), preterm newborns, and the elderly. However, the true burden of IFD may be underestimated because the currently available diagnostic methods are not sensitive enough and most fungal infections are not reportable diseases. *Candida* spp., *Aspergillus fumigatus* species complex, *Cryptococcus neoformans*, *Pneumocystis jirovecii*, endemic dimorphic fungi, and mucormycetes are the major fungal pathogens responsible for most IFDs. Additionally, since fungal species are constantly evolving, emergent fungal infections are often reported [1,2,3,4]. 

The most common strategy for fungal survival in the environment is the formation of spores. Because of their small diameter (~2.5–3.5 µm), spores can be transported great distances by normal atmospheric conditions such as convection currents and wind. Recent study recognized desiccated state and natural folding as pre-adaptations that support long-distance transport of viable cells through air. Natural folding was previously described in pollen to accommodate controlled and reversible water loss and was defined by the term ‘harmomegathy’. After settling on surfaces, spores can be agitated by different activities (cleaning, walking, etc.) and dispersed in the air again [5,6,7]. The slightest air current can cause spores to disperse due to their remarkable hydrophobicity, and these airborne conidia are protected from ultraviolet irradiation due to the melanin in their cell wall. The degree varies from mild to highly hydrophobic which impacts the efficiency of spore dispersibility. Conidial hydrophobicity is conferred by the surface hydrophobin encoded by the *rodA* gene. *A. fumigatus* species complex conidia are considerably more hydrophobic than those of other aspergilli such as *A. nidulans* species complex [8,9].

The first outbreak of invasive aspergillosis associated with construction and renovation works was described in 1976 by Aisner J. et al. Eight cases of invasive aspergillosis emerged in cancer patients after relocation of a hospital ward to a new built facility where dry fireproofing material was discovered as a source of *Aspergillus* spores [10]. Approximately half of healthcare-associated *Aspergillus* outbreaks are caused by construction or renovation activities within or around hospitals [11]. In the recent study by Viegas et al., *Aspergillus* spp. were observed in primary health care centers with the highest prevalence on floor surface swabs. The study intended to determine the prevalence of *Aspergillus* in the clinical environment through a novel multi-approach sampling protocol; both active and passive sampling methods were used in the study. Active sampling consisted of air sampling by two different methods (impaction and impinge collecting air samples of 600 L), and passive sampling methods comprised surface swabbing using a 10 by 10 cm square stencil (namely, floors, considered as the most critical surface), electrostatic dust clothes (EDCs) with a surface exposure area of 94.2 cm^2^, settled dust collected for 10 min at a minimum 0.75 m above floor level by a vacuum cleaner equipped with a 40 micron nylon mesh and the dust collected from the vacuum bag, and filters from heating, ventilation, and air conditioning (HVAC) systems. *A. niger* species complex and *A. versicolor* species complex were often detected in the clinical environment [12].

For healthy individuals, environmental exposure to etiological agents such as fungal spores results in no adverse effects. However, in immunosuppressed and at-risk patients, this exposure is a serious threat that can lead to IFD. Additionally, numerous fungal outbreaks have been reported in healthcare settings during construction activities, showing that prevention of these infections is an essential and necessary step when such activities are planned and performed to avoid unwanted consequences for patients hospitalized at that particular moment [2,7,13,14]. Scientific evidence about the efficacy and clinical relevance of specific infection control measures is still either lacking or conflicting, but the empirical evidence presented in the description of numerous outbreaks and recommendations to support these measures is constantly growing [13,14,15].

Therefore, the goal of this overview was to: (1) give insight into the recent data about the burden of IFDs associated with construction and renovation in healthcare settings; and (2) to overview recent evidence about available prevention and infection control measures.

## 2. Construction and Renovation in Healthcare Settings as a Source of Fungi

### 2.1. Permanent Need for Construction and Renovation

Modern medicine is constantly introducing novel technology and therapies. Construction and refurbishment are a constant occurrence in hospital facilities to keep up with the demands of contemporary healthcare. The objective is to increase patient care and safety, take into account community requirements, and provide more services, whether a new facility is being built or an existing one is being renovated. When it comes to hospital building and remodeling, more and more architects and designers are taking evidence-based results into consideration. In addition, improving the indoor environment in hospitals can cut down on the typical length of stay by about 11% [16,17].

Different types of works are recognized in the hospital environment including: (1) construction (build-up of a new healthcare facility); (2) renovation (works in an existing healthcare facility); (3) demolition (complete or partial tearing down of existing buildings and structures using controlled methods); and (4) excavation (movement of rock, dirt, and mud to create space for construction to begin; anything that has to do with tunneling through earthy materials and removing them falls under the category of excavation) [18].

### 2.2. How Construction Work Affects the Burden of Fungi and the Patients

Dust contamination and dispersion of large amounts of fungal spores makes hospital construction and renovation work an independent risk factor for fungal infections in immunosuppressed patients [19].

Large numbers of *Aspergillus* spores are liberated during construction or renovation activities. Because of their small diameter, the spores can reach small airways and alveoli, germinate to hyphae, and cause invasive disease in patients with risk factors [20]. Construction activities have been proven to be independent risk factors for other invasive fungal infections as well, namely mucormycoses, but invasive aspergillosis is the most common [21,22,23]. Nosocomial outbreaks involving *Scedosporium/Lomentospora* infections associated with a construction site at a hospital are described. The distinct characteristic of this fungal genus being highly antifungal resistant leading to high mortality with a generally fatal outcome despite antifungal treatment is also demonstrated in the description of outbreaks. In the first outbreak of disseminated *S. prolificans* infections occurring as a nosocomial outbreak associated with renovation inside a hospital described by Alvarez et al. in 1995, there were four clinical cases with positive blood cultures among neutropenic patients and all had fatal outcomes [24,25]. *Fusarium* infections as a part of outbreaks associated with excavation works were described, but this fungal genus was also isolated from hospital water distribution systems during periods of construction in some studies [25,26]. Construction and renovation works also add other diverse fungal types into the air. In a study by Abdel Hammed et al., *Cladosporium* followed by *Aspergillus* were the common fungal genera detected in air, although the clinical cases of IFD were not described [27].

## 3. Outbreaks of Invasive Fungal Infections Associated with Hospital Construction and Renovation

### 3.1. Burden of Outbreaks

**Figure 1:**
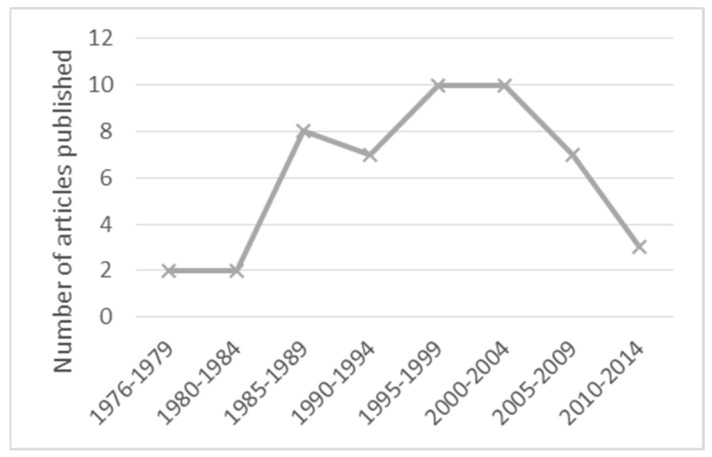
Outbreaks and infections associated with construction, renovation, or demolition described in published articles from 1976 to 2014. Author’s adaptation of [23].

In a review of outbreaks reported between 1966 and 2005, there were 53 affecting 458 patients and construction or demolition work was often (49.1%) considered to be the probable or possible source of the outbreak [6]. A review of outbreaks reported between 1976 and 2014 found 49 articles describing outbreaks of IFD associated with construction, renovation, and demolition. After the first described outbreak in 1976, the number of reported cases of outbreaks increased gradually with 7–10 articles in 5-year periods till 2009. In the period 2010–2014, a reduction in reported outbreaks was noted and the authors interpreted this as a consequence of improved infection control or publication bias (by authors or journals) given the large number of previously published outbreaks [23] (Figure 1).

In order to see the reports published after 2014 until the present, in this review a search was made of the literature in PubMed using the terms “fungi”, “aspergillus”, “mucor”, “mucorales”, “zygomycetes”, “scedosporium”, “lomentospora”, “fusarium”, and “cladosporium” in combination with the terms “construction” and “renovation” from 1 January 2015 to 27 December 2022. A web-based register of nosocomial epidemics “www.outbreak-database.com (accessed on 1 December 2022)” was also searched for outbreaks and infections due to any type of *Aspergillus* spp. [28]. Fungal outbreaks and infections associated with construction or renovation after 2014 until the present are summarized in Table 1.

Our search resulted in twelve reports focusing on IFD during construction or renovation works in the seven-year period from 2015 to 2022 showing a slightly increasing trend in comparison to the previous five-year period (2010–2014) described by Kanamori et al. when three studies were published [23]. According to country of origin, France and Turkey accounted for two studies each, and Australia, Brazil, Canada, Columbia, Germany, South Korea, Thailand, and Tunis accounted for one study each.

The settings in all twelve studies were university hospitals and populations investigated included mostly patients with hematologic malignancies except for three studies that also included other groups of immunosuppressed patients (solid organ transplant recipients, patients on corticosteroid therapy, HIV patients in advanced stage) and one study including COVID-19 intensive care unit (ICU) patients (Table 1). High risk populations for invasive fungal infections are traditionally considered to be patients with hematologic malignancies (especially neutropenic AML and ALL patients and recipients of allogeneic HSCT) and solid organ transplant recipients (especially heart and lung transplant recipients). Changes in invasive aspergillosis epidemiology has created new risk groups that should also be considered when planning prevention measures during construction or renovation works in the future. These groups include patients with severe influenza, chronic obstructive pulmonary disease receiving high dose corticosteroids, severe alcoholic liver cirrhosis, burns, patients in intensive care units, and patients with COVID [41,42,43,44,45]. In six studies, the type of fungi causing the IFD was determined—in five studies, *Aspergillus* spp. and *Mucor* spp. and in one study, *Lomentospora prolificans*. Outbreaks involving other fungal genera such as *Fusarium* and *Cladosporium* were not detected during this period.

Different preventive measures were applied in the studies published in the seven-year period from 2015 to 2022. Procedures intended to decrease and control dust contamination included:

- increased cleaning of surfaces and air conducts [29,33,40];
- closing or sealing of windows of rooms with at-risk patients [29,31,33];
- plastic barriers between inpatient-care areas and construction areas to prevent dust from entering [32,40];
- adhesive carpets for collecting dust at the entry ways of the unit [31];
- lock chambers between hospitalization units and building sites with temporary rigid plastic walls [31];
- permanent humidification (moistening of construction debris) of the location of demolition by constant blasting of water (water jets) during demolition [29,31];
- covering of rubble containers and quickly removing [31].

Pedestrian traffic for healthcare workers, patients, construction workers, and visitors was limited and redirected to avoid construction areas and limit the entrance of contaminated particles [29,31,33]. For immunosuppressed patients, N95 masks were recommended when going near construction work areas [33]. For neutropenic patients, surgical masks were required when they had to leave the room [31].

Patients were divided into risk groups and placed in the more protected environment if they were classified in the higher risk groups (patients with acute leukemia and recipients of hematopoietic cell transplantation (HCT)) [29]. For them, the use of rooms equipped with high-efficiency particulate air filtration (HEPA) and positive pressure was applied. Portable HEPA filters were used in two studies [32,38,40]. The rest of the patients were placed in conventional units [32,33,40].

Antifungal prophylaxis, namely posaconazole and micafungin, was administered in a few studies [32,38]. In one study, a combination of serum galactomannan and screening thoracic computed tomography, in addition to universal prophylaxis, was proposed [34].

Education included raising the awareness of healthcare workers about the mortality of invasive aspergillosis, especially in immunocompromised patients, and was also included in preventive measures [29,33].

In ten out of eleven studies, periodic monitoring of the quality of air was performed. Results of fungal concentrations may differ according to the methods used to collect air and environmental samples. Therefore, different methods used for the collection and cultivation of air and environmental samples, as well as for fungal identification, together with the results of airborne fungal levels in the studies during 2015–2022 period are shown in Table 2. In all studies in which air samples were collected, portable air samplers were used. They aspirate and inoculate airborne spores through a sampling grid onto growth medium, usually Sabouraud medium or some of its modifications [29,30]. The frequency of air sampling collection was different (weekly, once a month, after corrective measures, before the beginning of hospital building works, every week during the building works and at the end). In addition, the volume of air (500 L, 1000 L) and time (30 min in indoor areas, 5 min in outdoor areas, or 20 min) during which the volume was sampled varied among studies. Locations were also different—100 cm from the patient’s bed, 50 cm from the room entrance, 1m above ground level, bathroom, corridors, etc. The mean total and specific fungal concentrations were expressed as colony-forming units per cubic meter of air (CFU per m^3^) [29,30,40]. In some studies, surface samples were collected by the swabbing with a moist cotton swab of 25 cm^2^ of each of the following surfaces: bed, window, curtain, door wrist, nightstand, table, and cupboard [30]. In two studies, the impact of seasonal variations on the air sampling results was observed. Gheith et al. found the total fungal flora CFU counts significantly increased during summer and autumn, but there was no significant (*p* = 0.28) difference in *Aspergillus* spp. CFU counts according to the season [30]. Loschi et al. demonstrated that airborne spore concentrations varied according to the season and were the greatest during autumn [31].

In one study, air treatment systems and aspergillosis control measures were lacking. Referring to that, authors found it surprising that the incidence of invasive aspergillosis in their patients (9.9%) ranged among the lowest recorded rates as compared to the 6–15% incidence reported in previous studies [30].

A multidisciplinary team was introduced in one study assessing airborne fungal spore levels and systematically adjusted preventive measures and monitored their effectiveness with air sampling and clinical results [31,40].

### 3.2. Fungal Species Involved in Outbreaks

The causative pathogens of fungal outbreaks are usually *Aspergillus* species, including *A. fumigatus* species complex, *A. flavus* species complex, *A. terreus* species complex, and *A. niger* species complex. Other fungal species were involved occasionally and included species belonging to the following genera: *Mucor, Rhizopus, Candida, Trichosporon, Paecilomyces, Scedosporium, Fusarium,* etc. [24,25,46]. It is worth noticing that other microorganisms besides fungi should be considered as a cause of outbreak associated with hospital renovation. For example, an outbreak of intermittent peritoneal dialysis peritonitis was attributed to the external wall renovation with a predominance of *A. baumanii* [47].

In the context of outbreaks associated with construction or renovation, invasive aspergillosis (IA) is attracting the most attentions due to aspergillus spores being a major component of airborne particulate matter and also due to mortality rate. *A. fumigatus* spore concentrations in the environment are estimated to be between 1 and 100 conidia/m^3^. The average adult likely inhales more than 100 spores daily. IA carries a 50% mortality rate overall; however, mortality rates approach 100% if diagnosis is delayed or missed [1].

## 4. Infection Prevention and Control Measures

### 4.1. Infection Control Risk Assessment (ICRA)

#### 4.1.1. ICRA Precaution Matrix

Prior to any construction or renovation work, an infection risk assessment (ICRA) should be performed as advised in different guidelines for over 20 years. Since 1996, an ICRA has been required by the Facility Guidelines Institute’s (FGI’s) guidelines and in 2003, in guidelines issued by the Centers for Diseases Control and Prevention [48,49]. The American Society of Health Care Engineering (ASHE) clearly defined what should be included in the ICRA process and in 2022 released the latest version Infection Control Risk Assessment 2.0 Matrix of Precautions for Construction, Renovation and Operations, which is being called ICRA 2.0 [50].

ICRA is conducted by an interdisciplinary team including both medical and construction professionals to decide what level of risk mitigations and barrier precautions should be applied in a healthcare facility that will undergo construction or renovation. The membership of the team depends on the size and type of the works and should mandatorily include representatives of the healthcare facility management, construction project team, healthcare technical services, infection prevention and control team, and healthcare personnel from relevant clinical areas [51].

Firstly, the type of construction activity is defined on a scale from A to D according to the increasingly large quantities of dust it will generate, with A meaning inspection and noninvasive activities, and D meaning major demolition and construction activities. It is worth mentioning that following external demolition work, an increase in the airborne concentration of *Aspergillus* spores, which does not start to decline until about the fifth day and reaches its initial level on the eleventh day, has been reported [52]. Secondly, patient risk groups that will be affected are identified in a scale from low risk to highest risk, with low risk meaning non-patient care areas and highest risk meaning procedural, invasive, sterile support and highly compromised patient care areas such as transplant and intensive care units, transfusion services, all oncology units, operating theaters, etc. Finally, the patient risk group (low, medium, high, highest) is matched with the planned construction activity type (A, B, C, D) to define the class of precautions needed (I, II, III, IV, or V) or level of infection control activities required (Table 2) [50]. The ICRA process schematically shown in Table 3 is commonly known as the ICRA precaution matrix.

In the new ASHE version ICRA 2.0 released in 2022, an additional step assessing the potential risk to areas surrounding the construction or renovation site is included. The surrounding areas (unit below, above, lateral, behind, and in front) that will be affected should be identified, as should the type of impact that will occur (Table 4). If more than one risk group will be affected, a higher patient risk group should be selected when the abovementioned ICRA precaution matrix is completed [50].

#### 4.1.2. Precaution Classes

Using the abovementioned matrix, the level of precautions needed can be determined. Depending on the class selected, measures include dust control, including sealing off the construction site, debris removal and cleaning, relocation of high risk patients, ventilation systems including adequate air filtration, and avoiding unnecessary traffic [22,49,53].

They have a range from Class I, comprising noninvasive work, and Class II, comprising limited dust and invasive work following standard precaution procedures, to Class IV and V, which include extensive measures, for example, constructing critical barriers, maintaining negative pressurization of the entire workspace using HEPA exhaust, etc. (Table 5).

Briefly, there are three main novelties in the ICRA 2.0 version in comparison to the previous one regarding mitigation activities comprised by the specific class of precautions:

- an additional category (Class V) is formed because four classes limited the ability to properly address large-scale projects; namely, in addition to mitigation activities performed before and during work comprised by Class IV, Class V includes the need to construct an anteroom large enough for equipment staging, cart cleaning, and workers that must be constructed adjacent to the entrance of the construction work area, as well as the requirement for personnel to wear disposable coveralls at all times during Class V activities and that must be removed before leaving the anteroom;
- Class II must never be used for construction or renovation activities;
- the development of an ICRA process guide on how the ICRA should be implemented [50].

### 4.2. High-Efficiency Particulate Air (HEPA) Filters

A HEPA filter is defined as a high-efficiency particulate air filter with a 99.97% efficiency for removing particles ≥0.3 μm in diameter. HEPA filters have versatile applications in terms of the prevention of IFDs. In the case of construction and renovation, they are used for minimizing dust production; namely, they can be used to maintain clean surroundings by HEPA vacuuming but also to maintain negative pressurization of the workspace using HEPA exhaust air systems directed outdoors [50]. On the other hand, they are also used to create protected environments for high risk immunocompromised patients by minimizing fungal spore counts by filtration of incoming air [49].

Patients that ideally should be placed in a protective environment with HEPA filters according to currently available guidelines include at least allogeneic HSCT recipients [48,54]. Retrospective studies of the use of HEPA filtration units showed a reduction in the number of *Aspergillus* organisms in the air and a decrease in the risk of nosocomial *Aspergillus* infections in that group of patients [55,56,57]. Besides HEPA filters, protective environments also include directed room airflow, positive air pressure in a patient’s room in relation to the corridor, well-sealed rooms, and high (>12) air changes per hour. HEPA filtration leads to a significant decrease in the number of microorganisms in the air, whereas laminar air flow increases air change in the cleanest zone, which is why both measures are frequently combined [58,59,60,61,62]. However, there are no well-executed randomized or controlled trials about HEPA filter efficacy in protecting HSCT recipients, instead the reliance had to be placed on descriptive studies, reports of expert committees, or on the opinions of respected authorities. The results of meta-analyses showed somewhat ambiguous results but still suggest that patients with hematological malignancies with severe neutropenia or patients with bone marrow transplants receive some benefit if they are placed in a protected environment [63].

No recommendation can be made for routinely placing a recipient of autologous HSCT or solid organ transplant in a protected environment [38]. When assessing the effectiveness of the HEPA filtration on reducing treatment-related mortality in multiple myeloma patients receiving autologous stem cell transplantation, Tsai et al. found it didn’t affect 100-day mortality [64].

The protective environment regimen is expensive and is a social burden on patients. There are approaches proposing less rigorous isolation and infection control procedures or even home care of patients undergoing allogeneic HSCT [65,66]. Some authors when making recommendations on how to start an HSCT program in low and middle income countries suggest that inpatient rooms with laminar flow rooms and/or HEPA filters are not necessary in underprivileged circumstances [67]. Moreover, filters should be replaced regularly based on manufacturers’ recommendations, and when there is ongoing construction, filtration efficiency should be monitored frequently to best determine the appropriate time for replacement [57]. However, in a survey conducted by Styczynsky et al. that included 177 European Society for Blood and Marrow Transplantation (EBMT) centers from 36 countries, the authors indicated that 99.4% of patient rooms were equipped with HEPA filters, but only 48.6% of the center’s staff were aware of, and could confirm, the regular replacement of filters based on manufacturers’ recommendations [68].

Although filtration with HEPA was effective during normal conditions, their role as a preventive measure during construction or renovation works is still not completely clear. There are numerous studies showing their effect in decreasing the amount of fungal spores in air samples, as well as their influence on the incidence of invasive aspergillosis. In the study conducted by Özen et al., portable HEPA filters installed in patients’ rooms seemed to be effective in preventing IFD in particular subgroups of hematology patients during construction. The IFD-preventive effect of HEPA filters was most marked in acute lymphoid leukemia patients, especially during consolidation treatment and moderate neutropenia (1 to 14 days). HEPA filters did not appear to reduce the rates of IFD in non-neutropenic patients or in patients with >14 days of neutropenia, patients undergoing induction treatment, or in patients with either acute myeloid leukemia or non-acute leukemia (multiple myeloma, solid tumors, lymphoma, etc.) [33]. Nihtinen at al. evaluated the efficacy of HEPA filters in a bone marrow transplant unit during a hospital building renovation, and during a period of 12 weeks, they did not report any cases of invasive aspergillosis [69]. Similarly, in the study by Oren et al., during building construction, there were no cases of invasive aspergillosis among patients hospitalized in rooms with HEPA filters, while in patients without environmental protection, there was an incidence of invasive aspergillosis of 29% [62]. The recent studies also report about HEPA filters as protective measures during hospital construction or renovation [30,33,35] Surprisingly, there are also studies, although conducted 20 years ago or more, in which HEPA filters alone were unable to prevent the rise of *Aspergillus* contamination related to building renovation [52,59].

Although the efficiency and benefits of fixed HEPA filters is well proven, the benefits of portable HEPA filters are still inconclusive. In the study by Salam et al., portable HEPA filters were effective in the prevention of IA and their use was associated with a significant reduction (51%) in the incidence of IA [70]. In the study by Özen et al., portable HEPA filters were placed in the rooms of patients undergoing treatment for hematological malignancies because of large-scale construction taking place near the hematology clinic. A total of 413 patients were treated during this 1-year period and the rate of IFD was 9.0% in the control group versus 4.4% in the intervention group (*p* = 0.04), and in the neutropenic patients, the IFD rate decreased from 17.2 to 7.3% (*P* = 0.03) [32].

According to CDC recommendations, portable HEPA filters can be used in the prevention of IA. They can filter air at the rate of 300–800 ft^3^/min and can be used to temporarily recirculate air in rooms with no general ventilation, augment systems that cannot provide adequate airflow, and provide increased effectiveness in airflow. Many of the newer HEPA units are fairly quiet, with sound levels <40 dB. However, the benefits conferred by the portable units require appropriate maintenance and education of staff. In addition, the effectiveness of the portable unit for particle removal is dependent on the configuration of the room, the furniture and persons in the room, the placement of the units relative to the contents and layout of the room, and the location of the supply and exhaust registers or grilles. If portable, industrial-grade units are used, they should be capable of recirculating all or nearly all of the room air through the HEPA filter, and the unit should be designed to achieve the equivalent of ≥12 ACH [49]. HEPA filters certainly may help prevent fungal outbreaks when applied with other environment control measures, but further studies are needed to clarify the extent in which they, as specific control measures, contribute to preventing healthcare-associated fungal infections during construction and renovation.

### 4.3. Air Sampling

Air sampling for determining fungal spore levels is continuously a part of studies investigating IFDs during construction or renovation work. Microbiologic air sampling as well as environmental culture are not routinely recommended but may be useful and advisable in the inspection of ventilation performance after maintenance or cleaning procedures of airflow systems, construction activities, and as a part of epidemiologic investigation and research purposes [14,54].

Air sampling for quality control purposes is problematic because of a lack of uniform air-quality standards [49]. The main obstacles are the lack of standardization regarding sampling procedures including the appropriate volume, number and location of collection as well as the interpretation of the results. Furthermore, fungal spore levels vary greatly among different studies and threshold levels above which IFD outbreaks can be expected are still not defined [48]. In other words, there is no consensus on the cut-off value for designating fungal spore level as either safe or dangerous [37,62]. Paradoxically, fungal infections were reported despite no fungal growth by air sampling [53]. According to the standards of the Brazilian Ministry of Health and the National Health Surveillance Agency (ANVISA), the maximum recommended value—VMR—for fungal contamination is 750 CFU/m^3^ in air, and its quality should be frequently checked (usually every 6 months). Lower values have been found in Portugal, where the limit is 500 CFU/m^3^, and in Canada, where 150 CFU/m^3^ has been established as the limit value [71]. Limits for specific fungi, especially *Aspergillus* as an agent of the most common mold infection, should be defined also. In the studies published from 2015 to 2022, airborne total fungal levels in hospitals during outbreaks associated with construction and renovation varied widely from 5.60 to 1887.67 CFU/m^3^, *Aspergillus* levels from 0 to 30 CFU/m^3^ [29,30,31,32,33,34,35,36,37,38,39,40].

The result of each air sampling represents the spore levels in the air at that particular point in time and that can be influenced by many different factors such as indoor traffic (employees and visitors entering the facility), cleaning activities dispersing sedimented spores, temperature, time of day or year, relative humidity, and the performance of the air-handling system components. To be meaningful, air-sampling results must be compared with those obtained from other defined areas, conditions, or time periods [49]. Particle counts in a given area within the healthcare facility should be evaluated against counts obtained in a comparison area. Making rank-order comparisons between clean, highly filtered areas and dirty areas and/or outdoors is one way to interpret sampling results in the absence of air quality and action level standards. Barreiros et al., who studied the Aspergillus conidia concentration in corridors, rooms without filters, and in rooms with HEPA filters before and after the demolition of a hospital wing, showed that the higher concentration of *Aspergillus* spores was in non-protected areas (corridors, rooms without filters) while fungal concentration did not increase in rooms with HEPA filters [29].

**Figure 2:**
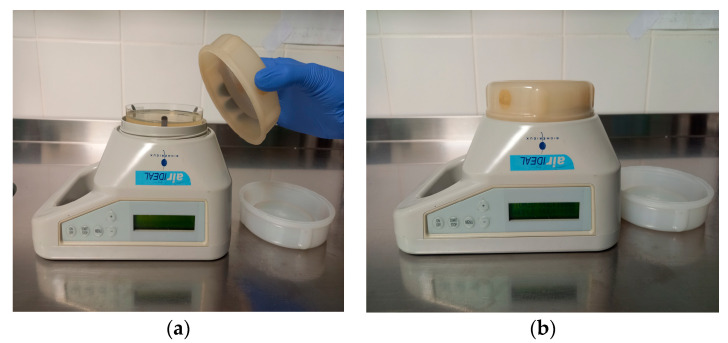
Air sampling with portable air sampler. (**a**) Preparing of air sampler for sampling procedure. Petri dish with Sabouraud medium is placed and covered with sterile sample grid. Time of duration and volume of air to be sampled is defined and visible on display. (**b**) Air sampling in process. Air containing spores is aspirated and inoculated through a sampling grid onto growth medium.

In healthcare, culture-based portable air samplers are the most practical for sampling bacteria, particles, and fungal spores because they can sample large volumes of air in relatively short periods of time (Figure 2). On the basis of the expected spore counts in the ambient air and the performance parameters of various types of volumetric air samplers, investigators of an *Aspergillus* outbreak have suggested that an air volume of at least 1000 L should be considered when sampling highly filtered areas [72]. The use of settle plates (i.e., the sedimentation or depositional method) is not recommended when sampling air for fungal spores because single spores can remain suspended in the air indefinitely [49,73,74]. Recently, electrostatic dust collectors (EDCs), an easily used passive sampling device used to collect settled dust, began to be more common in indoor air quality assessment. EDCs consist of electrostatic cloths that function as the surface area for dust sedimentation [75]. When using this passive method, only results by area or plate (CFU/m^2^/plate) can be obtained, whereas a small number of existing guidelines and published literature deal in CFU/m^3^, making the results difficult to compare. The main advantage of this method is that it can collect the sample from a larger period of time (weeks to several months), whereas air samples can only reflect the load from a shorter period of time (mostly minutes) [76]. There are studies that evidence the importance to perform in parallel active methods with air samplers and passive methods, since active methods provide information about the contamination load while passive methods such as EDCs give more information regarding occupational exposure to bioaerosols. According to this, EDCs give a more complete occupational exposure scenario regarding bioaerosols, allowing a more rigorous assessment, but their use in the assessment of airborne fungal levels in the context of infection prevention and control in healthcare facilities has only started and needs to be further investigated [12,77,78].

A recent study investigating the relationship between airborne fungal contamination and the incidence of invasive aspergillosis during construction periods in a tertiary care hospital showed that the incidence of invasive aspergillosis was higher in periods when airborne fungal spore levels tended to be higher (2.35 CFU/1000 L) [37]. Additionally, this study showed the correlation between specific construction types, airborne fungal contamination, and the incidence of invasive aspergillosis; namely, demolition and excavation works seemed to disperse larger amounts of fungal spores than other types of construction works and may contribute to the increased incidence of invasive aspergillosis [37]. Barreiros et al. showed that implosion of the building resulted in a great increase in the concentration of fungi in the air, while mechanical demolition caused the increase to be restricted to the less protected areas. However, in their study, the incidence of invasive aspergillosis did not increase [27]. As opposed to that, Wirmann et al. described extensive demolition works without a difference in mean concentrations of *A. fumigatus* species complex spores between the three periods before (17.5 CFU/m³), during 30 (20.8 CFU/m³) (*p* = 0.26), and after demolition (17.7 CFU/m³) (*p* = 0.33), as well as no significant difference in invasive aspergillosis cases between these periods. These results were interpreted as being a consequence of successfully implemented preventive measures [36].

Previous studies indicated the possible association of meteorological effects on the dynamics of *Aspergillus* spp. spores in the air. However, these findings are conflicting. In some studies, meteorological data did not correlate significantly with the airborne *A. fumigatus* species complex spore concentration [36]. As opposed to that, Pilmis et al. showed that elevated *Aspergillus* spp. concentration was associated with higher temperature and suggested that demolition work should be performed during the winter and fall season [79]. The recent study by van Rhijn et al. also suggests that airborne *Aspergillus fumigatus* species complex spores were more abundant during the summer months, which appeared to be driven by increased temperatures and lower wind speeds [80]. Other studies from other countries support higher airborne fungal concentrations in summer or autumn than in winter [81,82,83]. In addition, some studies showed differences in climate can influence even the number of invasive aspergillosis cases. In a recent Spanish study, a higher incidence of invasive aspergillosis was observed in the months with higher humidity and rainfall [84]. Climate factors should be considered when interpreting air sampling results obtained in different time periods.

### 4.4. Do FFP2 Masks Have Protective Roles for Immunocompromised Patients during Construction and Renovation Works?

Immunocompromised patients need to be segregated from construction or renovation works. Air filtration is mainly limited to the patients’ rooms. The problem of inhalation of *Aspergillus* spores outside these rooms, mainly on transport for diagnostic procedures or in an outpatient setting, is still not resolved.

In outbreaks associated with construction and renovation works from 2015 to 2022, surgical masks were required for neutropenic patients when they had to leave their room [31]. In addition, N95 masks were recommended for immunocompromised patients when going near construction work areas [33]. Surgical masks are routinely used by high risk patients in many institutions, but their benefit for these patients has never been shown clinically [85]. According to CDC guidelines on the prevention of nosocomial pneumonia, the length of time that immunocompromised patients in protective environments are outside their rooms for diagnostic procedures and other activities should be minimized, and severely immunocompromised patients should be instructed to wear a high-efficiency respiratory protection device (e.g., an N95 respirator) when they leave their protective environment during periods when construction, renovation, or other dust-generating activities are ongoing in and around the healthcare facility. However, this recommendation is not supported by a properly designed clinical study. Furthermore, whether and what type of respiratory protection device (e.g., surgical mask, N95 respirator) severely immunocompromised patients should wear when leaving their protective environment during periods when there is no construction, renovation, or other dust-generating activity in progress in or around the healthcare facility is not specified [65].

However, according to recent ESCMID-ECMM-ERS guidelines published by Ullmann et al., protective masks for patients are proven not to be effective outside the protected area with strength of recommendation CII (meaning “societies marginally support a recommendation for use”) [54]. The recommendation is based on the prospective, randomized, multicenter study investigating efficacy and tolerability of the well-fitting FFP2 mask conducted by the Infectious Diseases Working Party (AGIHO) of the German Society of Hematology and Oncology. Among the 41 patients undergoing intensive myelosuppressive chemotherapy for acute leukemia or allogeneic hematopoietic stem cell transplantation during construction works in the hospital and assigned to wear well-fitting FFP2 masks without interruption whenever the room was left, not even a slight trend to less invasive aspergilloses could be observed [86].

### 4.5. Antifungal Prophylaxis

As diagnostic methods for IFD are imperfect, for patients known to be at high risk for IA, especially patients with profound and prolonged neutropenia or with active graft-versus-host disease (GvHD), primary prophylaxis is recommended by the current guidelines [54,87].

Several studies showed doubtful impacts of antifungal prophylaxis on the prevention of invasive aspergillosis during construction works. Combariza et al. showed that the addition of prophylaxis with posaconazole to environmental control measures led to a decrease in the incidence of invasive aspergillosis from 14.4% to 6.3 % [34]. Le Clech et al. showed that the incidence density of IA significantly decreased during construction periods when posaconazole prophylaxis was used (1.59 vs. 4.87 per 100 hospitalization days, *p* < 0.0001) and suggested the interest of antifungal prophylaxis in addition to HEPA filtration in the prevention of IA during hospital building works [38]. The study by Oren et al. described the experience with amphotericin B prophylaxis during building construction when the incidence of invasive aspergillosis declined marginally from 50% to 43% [62]. A cost-effectiveness study on interventions for the prevention of IA during hospital construction revealed that the addition of antifungal prophylaxis to environmental control measures led to higher costs. However, it was shown to be more effective than environmental measures alone [88]. In the study by Chabrol et al., invasive aspergillosis was diagnosed in 12% of patients in the non-prophylactic group and 4.5% in the prophylaxis group with voriconazole or caspofungin [89]. Chang et al. registered no invasive aspergillosis when infection control measures (barriers, face-masking) were applied simultaneously with voriconazole prophylaxis [53]. In summary, the value of antifungal prophylaxis in the prevention of invasive aspergillosis associated with construction works in healthcare settings is difficult to assess because other preventive measures (dust control, HEPA filters, face masking) were simultaneously applied as well.

Clinical risk assessment is the basis for the consideration of prescribing antifungal prophylaxis in selected high risk group patients. Its use in association with mechanical protection is not clearly recommended during hospital construction/demolition works. However, in the event of a possible outbreak of aspergillosis in a patient group not belonging to forementioned high risk groups, antifungal prophylaxis should be considered and expert advice sought [90].

## 5. Conclusions

Insight into the recent studies describing IFD outbreaks associated with construction or renovation shows that the number of these studies is on the rise again. Applying adequate prevention measures is still a challenge, not just for healthcare workers but also for architects and construction workers as well. The role of multidisciplinary teams in the planning and monitoring of prevention measures cannot be overemphasized. Dust control is an inevitable part of every prevention plan. HEPA filters certainly may help prevent fungal outbreaks when applied with other control measures, but further studies are needed to clarify the extent in which they, as a specific control measure, contribute to the prevention of healthcare-associated fungal infections during construction and renovation. Airborne fungal spores should be monitored during construction in hospitals with immunocompromised patients, but further studies are needed to define a cut-off value for a “threating” level of fungal spore contamination. Additionally, climatic factors should be taken into account in each region or hospital when interpreting the results of air sampling. The value of antifungal prophylaxis in the prevention of invasive aspergillosis associated with construction works in healthcare settings is difficult to assess because other preventive measures (dust control, HEPA filters, face masking) in the majority of described outbreaks were simultaneously applied as well. Although official recommendations exist, they are still not based on randomized clinical trials but on few meta-analyses, a large number of descriptive reports, and the opinion of respective authorities. Outbreak reports in the literature are a valuable resource and should be used for educational purposes as well as for preparing outbreak investigations. Further studies are needed for a better understanding of the disease itself and improved prevention strategies. They will help to choose and apply the most appropriate measures and reduce incidence and mortality in high risk patients.

## Figures and Tables

**Table 1:** Characteristics of fungal outbreaks and infections associated with construction or renovation described in articles published from January 2015 to December 2022.

Author, Year	Patient Population	No. of Patients Infected	No. of Patient Deaths	Type of Infection (Site)	Type of Fungi	Reservoir or Source	Molecular Typing	Control Measures
Barreiros, G. et al., 2015 [29]	Patients divided into three risk groups: the highest—acute leukemia, HCT recipients; intermediate—HIV in advanced stage, high dose corticosteroids therapy, SOT recipients; low—all other patients	Incidence of IA (cases per 1000 admissions): 0.9 in the 12 months before demolition, 0.4 during demolition; and 0.5 in the 12 months after implosion	not specified	Invasive aspergillosis (sites not specified)	not specified	Mechanical demolition to detach the two wings and implosion	not performed	Permanent humidification, limitation of circulation of people in areas close to demolition, sealing of the windows, increase in environmental cleaning, staff education, rooms with HEPA filters for highest risk patients, high risk patients used N95 masks when circulated in non-protected areas
Gheith, S. et al., 2015 [30]	Hematologic malignancies (AML, ALL); patients with a profound postchemotherapy neutropenia were included	11	not specified	Invasive aspergillosis (lung) except one patient with invasive ethmoditis with periorbital expansion and without prior lung involvement	*A. niger* species complex, *A. flavus* species complex, *A. nidulans* species complex, *A. fumigatus* species complex	Airborne contamination was a significant and independent IA risk factor in the renovation work setting	not performed	Air treatment systems and aspergillosis control measures were lacking in the hospital
Loschi, M. et al., 2015 [31]	Hematologic malignancies (AML, ALL, NHL, HL, multiple myeloma, MDS, CLL)	102	8	Invasive pulmonary aspergillosis except one disseminated with cerebral lesions	unknown	Five years of indoor and outdoor renovations, including excavations, collapsing of walls, sanding, and wiring	not performed	Air lock chambers between hospitalization units and building sites’ adhesive carpets for collecting dust, surgical masks required for neutropenic patients when leaving their rooms, windows were sealed, pedestrian traffic rearranged
Özen, M. et al., 2016 [32]	Hematologic malignancies (AML, ALL)	29	not specified	Invasive fungal infections (site of infections not described)	unknown	Source of infection was not revealed; large-scale construction taking place near the hematology clinic probable source of infection	not performed	Portable HEPA filters were installed in patients’ rooms
Combariza, J. et al., 2017 [33]	Hematologic malignancies (AML, ALL)	29	not specified	Invasive aspergillosis (site of infections not defined)	unknown	Outbreak was associated with an extensive building work	not performed	Dust control procedures (cleaning, sealing of rooms), plastic barriers, redirection of traffic, HEPA filters and positive pressure, FFP2 masks for patients when going near construction area, prophylaxis with posaconazole
Kabbani, D. et al., 2018 [34]	Heart transplant recipients	7	3 (in one patient non-IA-related death and in one patient IA- and mucormycosis-related death)	Invasive aspergillosis (lung)	*A. fumigatus* species complex	Air and environmental sampling failed to reveal source of infection; the construction around the hospital might have played a role	not performed	Screening chest CT scans; all new HTRs antifungal prophylaxis with micafungin intravenously daily during hospitalization, followed by inhaled amphotericin 20 mg twice a day for 3 months
Wirmann, L. et al., 2018 [35]	Immunocompromised patients (hematologic malignancies, solid organ transplant recipients)	44	not specified	Invasive aspergillosis	not specified	Extensive demolition works	Genotyping by microsatellite PCR of the azole-resistant environmental and clinical isolates showed a polyclonal distribution	Water jets to suppress dust emission, all windows facing the demolition site closed; immunocompromised patients leaving the protective area were equipped with high-efficiency filtration face masks; patients undergoing bone marrow transplantation are located in rooms protected by high-efficiency particulate air (HEPA) filters and positive pressure
Park, J.H. et al., 2019 [36]	Hematologic malignancies (AML the most common)	29	8	Two patients with invasive aspergillus sinusitis and remaining patients with invasive pulmonary aspergillosis	*A. fumigatus* species complex, *A. flavus* species complex, *A. niger* species complex	Radiotherapy facility construction	not performed	HEPA filters and positive pressure ventilation systems
Boan, P. et al., 2020 [37]	Hematologic malignancies (AlloHCT, CLL, AML)	4	4	Soft tissue and disseminated infections	*Lomentospora* *prolificans*	Environmental source was not found; minor earthworks adjacent to the hospital and water leakage from plumbing at a distant part of the hospital are mentioned	WGS suggested the infections were not caused by a single strain	no change in practice
Le Clech, L. et al., 2020 [38]	Hematologic malignancies (AML)	8	not specified (one-year survival for patients with IA was 60%)	Invasive pulmonary aspergillosis	not specified	Air samples revealed *A. fumigatus* species complex and *A. versicolor* species complex; periods of hospital construction, renovation and demolition near the Department of Clinical Hematology	not performed	HEPA filters
Atilla, A. et al., 2022 [39]	Hematologic malignancies (AML, lymphoma, ALL, MDS, multiple myeloma)	412 patients with invasive fungal infection and 145 patients with invasive mold infection	136 (28-day mortality 33.0%)	Invasive aspergillosis (lung), mucormycosis, and invasive candidosis (blood culture)	*A. flavus* species complex, *A. fumigatus* species complex, *A. terreus* species complex, *Mucor* spp.	Source of infection was not revealed; proximity of construction site probable source of infection	not performed	Relocation of patients to the new hospital building
Sathitakorn, O. et al., 2022 [40]	COVID−19 ICU patients	4	4	Invasive pulmonary aspergillosis	*A. flavus* species complex, *A. fumigatus* species complex	Renovation and construction activity near the ICU (demolition work including removing the floor covering, ceiling tiles, case work, and new wall construction on the same floor)	not performed	Site containment, installation of critical barriers to seal construction areas from clinical areas, cleaning of construction areas, use negative air-pressure handling of the construction site using exhaust fans, and installing portable HEPA filters at the construction site

AML—acute myeloid leukemia; ALL—acute lymphoblastic leukemia; CT—computed tomography; CLL—chronic lymphocytic leukemia; HEPA—high-efficiency particulate air; HTR—heart transplant recipient; HL—Hodgkin lymphoma; HCT—hematopoietic cell transplantation; IA—invasive aspergillosis; ICU—intensive care unit; MDS—myelodysplastic syndrome; NHL—non-Hodgkin lymphoma; SOT—solid organ transplantation; WGS—whole genome sequencing.

**Table 2:** Methods used for collecting air and environmental samples, fungal identification, and measured total and specific airborne fungal levels during fungal outbreaks and infections associated with construction or renovation described in articles published from January 2015 to December 2022.

Author, Year	Air Samples Collection	Environmental Samples Collection	Fungal Identification	Airborne Total Fungal Level	Airborne Specific Fungal Level	Environmental Samples Results
Barreiros, G. et al., 2015 [29]	Using a 6-stage Andersen air sampler (Andersen: Thermo Fisher Scientific, Inc. Waltham, MA, USA) which collects air at a rate of 28.3 L/min; each stage of the air sampler was filled with 90 × 15 mm plates containing 2% Sabouraud dextrose agar (DIFCO, Houston, TX, USA) with gentamycin (200 ug/mL). The samples were performed during 30 min in indoor areas and 5 min in outdoor areas. After sampling, the plates were incubated at 25 °C for at least 7 days.	not performed	Colony counts were performed weekly, and subcultures were made using potato dextrose agar (DIFCO), Czapek agar (DIFCO), lactrimel agar, oat agar, and malt extract agar. Identification of fungi was performed on the basis of morphological parameters, initially by observing the characteristics of colonies in a stereoscopic microscope. Subcultures were performed if the colonies had the characteristics consistent with clinically relevant fungi (*Aspergillus, Fusarium*, agents of mucormycosis, as well as *Cladosporium* and *Penicillium).* The most frequent species was *A. niger* complex.	The concentration increased with values of 148.17 CFU/m^3^ in the historical period, 271.45 CFU/m^3^ during demolition, 1887.67 CFU/m^3^ on the day of implosion and 204.10 CFU/m^3^ in the postimplosion period.	Concentration of *Aspergillus* spp. varied significantly: 3.71 CFU/m^3^ in the historical period, 7.18 CFU/m^3^ in the demolition period, 22.5 CFU/m^3^ on the day of implosion, and 15.13 CFU/m^3^ of air in the postimplosion period. The mean concentration of agents of mucormycosis was low in all periods (0.2 CFU/m^3^ in the historical period, zero in the periods of mechanical demolition and implosion, and 0.1 CFU/m^3^ in the postimplosion period. Dematiaceous fungi other than *Cladosporium* spp. were encountered in small amounts (mean 3.45/m^3^).	N/A
Gheith, S. et al., 2015 [30]	Air samples were collected at 100 cm from the patient’s bed and at 50 cm from the room entrance by using a Microflow portable air sampler (Aquaria Srl, Lacchiarella, Italy) that aspirates and inoculates airborne spores through a sampling grid onto the Sabouraud chloramphenicol medium.	Surface samples were collected by the swabbing with a moist cotton swab of 25 cm^2^ of each of the following surfaces: bed, window, curtain, door wrist, nightstand, table, and cupboard. Each sample was inoculated onto the Sabouraud–chloramphenicol medium (Bio-Rad).	Fungal colonies were identified and counted after a 5-day incubation at 27 °C. The identification of the filamentous fungi was based on both the macroscopic and microscopic characteristics of the colonies. Aspergilli were identified to the section level because morphological differentiation of species within the same section is questionable in a routine laboratory setting. Colony-forming units (CFU) were expressed per m^2^ and per m^3^ in surface and air samples, respectively.	In air samples, the total fungal contamination (CFUs) significantly correlated with *Aspergillus* spp., *Aspergillus* section *Nigri* and *Aspergillus* section *flavi.*	*Aspergillus* spp. CFU counts in air samples during 14 months of renovation was higher (8.1 vs 6 mean CFU/m^3^, *p* = 0.031) than during the period after work stopped.	The same pattern was observed (12.34 vs 6.8 mean CFU/m^3^, *p* = 0.00002) regarding *Aspergillus* CFU counts in surface samples.
Loschi, M. et al., 2015 [31]	Airborne *Aspergillus* concentrations were measured weekly by repeated air sampling in each department. Areas with positive samples were retested after corrective measures. Samples were collected using a Reuter Centrifugal Impaction (RCS) High Flow Air Center (Biotest Hycon, Germany) loaded with ready-to-use culture media on flexible agar strips with modified Sabouraud dextrose agar for yeast and molds, γ-irradiated in double wrapper to determine the total number of fungal spores in the air (Biotest Hycon, Germany).	not performed	When the cultures were positive, colonies were quantified as colony-forming units (CFU) per cubic meter (m^3^). Less than 20 CFU/m^3^ was considered acceptable in all areas except for ICU where 0 CFU/m^3^ was required.	not performed (only *Aspergillus* concentrations were measured)	Airborne spore levels ranged from 0 to 30 CFU/m^3^. There was an increased number of positive samples in the standard unit compared to the ICU (*p* < 0.0001).	N/A
Özen, M. et al., 2016 [32]	The level of airborne particulates in patients’ rooms to evaluate HEPA filter efficiency was randomly measured; the methods was not specified.	not performed	not specified	The levels of particulates in the patients’ rooms were within acceptable limits; the results were not specified.	not specified	N/A
Combariza, J. et al., 2017 [33]	not performed	not performed	N/A	N/A	N/A	N/A
Kabbani, D. et al., 2018 [34]	not specified	not specified	not specified	not specified; air sampling of potential common pre- and post-admission exposure links during this outbreak failed to reveal a common environmental source of infection.	not specified	not specified; environmental sampling of potential common pre- and post-admission exposure links during this outbreak failed to reveal a common environmental source of infection.
Wirmann, L. et al., 2018 [35]	The measuring apparatus MAS−100 (Merck Chemicals GmbH, Darmstadt, Germany) was used. The apparatus was placed 1m above ground level. For each sample, 500 L of air was collected on malt extract agar plates. The measuring head of MAS−100 was autoclaved between each sampling day. The agar plates were incubated at 50 °C for 48 h.	not performed	Samples that showed visible mold growth after the incubation period were examined microscopically to identify *A. fumigatus*. All *A. fumigatus* isolates were plated on to Sabouraud dextrose agar containing 4mg/L itraconazole to screen for azole resistance. Grown isolates were subjected to antifungal susceptibility testing for itraconazole, voriconazole, posaconazole, and isavuconazole, according to EUCAST standard 9.3. The cyp51A gene was sequenced for all isolates with elevated minimum inhibitory concentration against an azole, as described recently. All colonies identified microscopically as *A. fumigatus* were counted and documented as colony-forming units (CFU)/m^3^. Additionally, the number of colonies was corrected according to Feller.	not performed (only *Aspergillus* spp. concentrations were measured)	Mean concentrations *of A. fumigatus* spores did not differ significantly be-tween the three periods before (17.5 CFU/m³), during 30 (20.8 CFU/m³) (*p* = 0.26), and after demolition (17.7 CFU/m³) (*p* = 0.33).	N/A
Park, J.H. et al., 2019 [36]	Air sampling was conducted once a month in the three hematologic wards during the construction period. A total of 1000 L of air was collected three times every 20 min by using a portable air sampler (AirPort MD8, Sartorius AG, Germany) located at each nurse station. Air was plated onto Sabouraud dextrose agar and incubated at 30 °C for five days.	not performed	After incubation, colonies were counted, and the data expressed as median colony-forming units (CFU) per 1000 L of air. Colonies were identified at the genus level based on macroscopic and microscopic findings (lactophenol cotton blue-stained preparation).	The total mold spore level tended to be lower in period 2 (5.60 CFU/1000 L) with lighter works such as framing, interior designing, plumbing, and finishing in comparison to period 1 (9.95 CFU/1000 L) with heavier works such as demolition and excavation.	Aspergillus spore levels were also lower in period 2 (1.70 CFU/1000 L) than in period 1 (2.35 CFU/1000 L).	N/A
Boan, P. et al., 2020 [37]	One cubic meter of air for fungal culture was sampled in affected patients’ rooms, other areas of the hematology wards, the cancer outpatient center, the main hospital concourse, car park, and open grounds.	Swabs for fungal culture were taken from patient sinks and various other surfaces of patients’ rooms in the hematology wards.	not specified	not specified	*Lomontospora prolificanswas* was not found in air samples.	*Lomontospora prolificans* was not found in environmental samples.
Le Clech, L. et al., 2020 [38]	Air sampling was conducted with the MAS−100 biocollector (Merck, Darmstadt, Germany) using Sabouraud chloramphenicol plates.	Surface samples were collected using a biocontact applicator (Oxoid, Dardilly, France).	not specified	not specified (results were presented as percentage of positive samples)	*A. fumigatus* species complex and *A. versicolor* species complex were detected; level not specified (results were presented as a percentage of positive samples).	*A. fumigatus* species complex and *A. versicolor* species complex were detected; level not specified (results were presented as a percentage of positive samples).
Atilla, A. et al., 2022 [39]	not performed	not performed	N/A	N/A	N/A	N/A
Sathitakorn, O. et al., 2022 [40]	not specified	not performed	not specified	At the front of ICU, the nursing station, the index patient anterooms, and rooms, airborne fungal bioburdens from air sampling were 235–290 CFU/m^3^ at all sites; the baseline standard airborne fungal bioburden was <150 CFU/m^3^ for the ICU.	not specified	N/A

ICU—intensive care unit; N/A—not applicable.

**Table 3:** ICRA precaution matrix—class of precautions according to construction type and patient risk group. Author’s adaptation of [50].

	Construction Type			
Patient Risk Group	Type A	Type B	Type C	Type D
Low risk group	I	II	II	III
Medium risk group	I	II	III	IV
High risk group	I	III	IV	V
Highest risk group	III	IV	V	V

I–V—precaution classes.

**Table 4:** Surrounding area assessment. Author’s adaptation of [50].

Unit Below:	Unit Above:	Unit Lateral:	Unit Behind:	Unit in Front:
Risk Group:	Risk Group:	Risk Group:	Risk Group:	Risk Group:
Contact:	Contact:	Contact:	Contact:	Contact:
Phone:	Phone:	Phone:	Phone:	Phone:
**Additional Controls:** ▫ noise▫ vibration ▫ dust control ▫ ventilation ▫ pressurization ▫ vertical shafts ▫ elevators/stairs **Systems impacted:** ▫ data ▫ mechanical ▫ med. gases ▫ hot/cold water	**Additional Controls:** ▫ noise ▫ vibration ▫ dust control ▫ ventilation ▫ pressurization ▫ vertical shafts ▫ elevators/stairs **Systems impacted:** ▫ data ▫ mechanical ▫ med. gases ▫ hot/cold water	**Additional Controls:** ▫ noise ▫ vibration ▫ dust control ▫ ventilation ▫ pressurization ▫ vertical shafts ▫ elevators/stairs **Systems impacted:** ▫ data ▫ mechanical ▫ med. gases ▫ hot/cold water	**Additional Controls:** ▫ noise ▫ vibration ▫ dust control ▫ ventilation ▫ pressurization ▫ vertical shafts ▫ elevators/stairs **Systems impacted:** ▫ data ▫ mechanical ▫ med. gases ▫ hot/cold water	**Additional Controls:** ▫ noise ▫ vibration ▫ dust control ▫ ventilation ▫ pressurization ▫ vertical shafts ▫ elevators/stairs **Systems impacted:** ▫ data ▫ mechanical ▫ med. gases ▫ hot/cold water

**Table 5:** Precaution Classes I–V before and during work activity according to Infection Control Risk Assessment (ICRA (2.0). Author’s adaptation of [50].

Precaution Class	Mitigation Activities
I	1. Perform noninvasive work activity as to not block or interrupt patient care. 2. Perform noninvasive work activities in areas that are not directly occupied with patients. 3. Perform noninvasive work activity in a manner that does not create dust. 4. Immediately replace any displaced ceiling tile before leaving the area and/or at end of noninvasive work activity.
II	1. Perform only limited dust work and/or activities designed for basic facilities and engineering work. 2. Perform limited dust and invasive work following standing precaution procedures approved by the organization. 3. This class of precautions must never be used for construction or renovation activities.
III	1. Provide active means to prevent airborne dust dispersion into the occupied areas. 2. Means for controlling minimal dust dispersion may include hand-held HEPA vacuum devices, polyethylene plastic containment, or isolation of work area by closing room door. 3. Remove or isolate return air diffusers to avoid dust from entering the HVAC system. 4. Remove or isolate the supply air diffusers to avoid positive pressurization of the space. 5. If work area is contained, then it must be neutrally to negatively pressurized at all times. 6. Seal all doors with tape that will not leave residue. 7. Contain all trash and debris in the work area. 8. Nonporous/smooth and cleanable containers (with a hard lid) must be used to transport trash and debris from the construction areas. These containers must be damp wipe cleaned and free of visible dust/debris before leaving the contained work area. 9. Install an adhesive (dust collection) mat at entrance of contained work area based on facility policy. Adhesive mats must be changed routinely and when visibly soiled. 10. Maintain clean surroundings when area is not contained by damp mopping or HEPA vacuuming surfaces.
IV	1. Construct and complete critical barriers meeting NFPA 241 requirements including: barriers must extend to the ceiling or, if ceiling tile is removed, to the deck above, and all penetrations through the barrier shall meet the appropriate fire rating requirements. 2. All (plastic or hard) barrier construction activities must be completed in a manner that prevents dust release. Plastic barriers must be effectively affixed to the ground and ceiling and secure from movement or damage. Apply tape that will not leave a residue to seal gaps between barriers, ceiling, or floor. 3. Seal all penetrations in containment barriers, including floors and ceiling, using approved materials (UL schedule firestop if applicable for barrier type). 4. Containment units or environmental containment units (ECUs) approved for Class IV precautions in small areas totally contained by the unit and that have HEPA-filtered exhaust air. 5. Remove or isolate return air diffusers to avoid dust entering the HVAC system. 6. Remove or isolate the supply air diffusers to avoid positive pressurization of the space. 7. Negative airflow pattern must be maintained from the entry point to the anteroom and into the construction area. The airflow must cascade from outside to inside the construction area. The entire construction area must remain negatively pressurized. 8. Maintain negative pressurization of the entire workspace by use of HEPA exhaust air systems directed outdoors. Exhaust discharged directly to the outdoors that is 25 feet or greater from entrances, air intakes, and windows does not require HEPA-filtered air. 9. If exhaust is directed indoors, then the system must be HEPA filtered. Prior to start of work, HEPA filtration must be verified by particulate measurement at no less than 99.97% efficiency and must not alter or change airflow/pressure relationships in other areas. 10. Exhaust into shared or recirculating HVAC systems, or other shared exhaust systems (e.g., bathroom exhaust) is not acceptable. 11. Install device on exterior of work containment to continually monitor negative pressurization. To assure proper pressure is continuously maintained, it is recommended that the device(s) has (have) a visual pressure indicator. 12. Contain all trash and debris in the work area. 13. Nonporous/smooth and cleanable containers (with a hard lid) must be used to transport trash and debris from the construction areas. These containers must be damp wipe cleaned and free of visible dust/debris before leaving the contained work area. 14. Worker clothing must be clean and free of visible dust before leaving the work area. HEPA vacuuming of clothing or use of cover suits is acceptable. 15. Workers must wear shoe covers prior to entry into the work area. Shoe covers must be changed prior to exiting the anteroom to the occupied space (non-work area). Damaged shoe covers must be immediately changed. 16. Install an adhesive (dust collection) mat at the entrance of the contained work area based on facility policy. Adhesive mats must be changed routinely and when visibly soiled. 17. Consider collection of particulate data during work to monitor and ensure that contaminates do not enter the occupied spaces. Routine collection of particulate samples may be used to verify HEPA filtration efficiencies.
V	1. Construct and complete critical barriers meeting NFPA 241 requirements including: barriers must extend to the ceiling, or if ceiling tile is removed, to the deck above, and all penetrations through the barrier shall meet the appropriate fire rating requirements. 2. All (plastic or hard) barrier construction activities must be completed in a manner that prevents dust release. Plastic barriers must be effectively affixed to the ground and ceiling and secure from movement or damage. Apply tape that will not leave a residue to seal gaps between barriers, ceiling, or floor. 3. Seal all penetrations in containment barriers, anteroom barriers, including floors and ceiling using approved materials (UL schedule firestop if applicable for barrier type). 4. Construct anteroom large enough for equipment staging, cart cleaning, and workers. The anteroom must be constructed adjacent to the entrance of the construction work area. 5. Personnel will be required to wear disposable coveralls at all times during Class V work activities. Disposable coveralls must be removed before leaving the anteroom. 6. Remove or isolate return air diffusers to avoid dust entering the HVAC system. 7. Remove or isolate the supply air diffusers to avoid positive pressurization of the space. 8. Negative airflow pattern must be maintained from the entry point to the anteroom and into the construction area. The airflow must cascade from outside to inside the construction area. The entire construction area must remain negatively pressurized. 9. Maintain negative pressurization of the entire workspace using HEPA exhaust air systems directed outdoors. Exhaust discharged directly to the outdoors that is 25 feet or greater from entrances, air intakes, and windows does not require HEPA-filtered air. 10. If exhaust is directed indoors, then the system must be HEPA filtered. Prior to start of work, HEPA filtration must be verified by particulate measurement at no less than 99.97% efficiency and must not alter or change airflow/pressure relationships in other areas. 11. Exhaust into shared or recirculating HVAC systems, or other shared exhaust systems (e.g., bathroom exhaust) is not acceptable. 12. Install device on exterior of work containment to continually monitor negative pressurization. To assure proper pressure is continuously maintained, it is recommended that the device(s) has (have) a visual pressure indicator. 13. Contain all trash and debris in the work area. 14. Nonporous/smooth and cleanable containers (with a hard lid) must be used to transport trash and debris from the construction areas. These containers must be damp wipe cleaned and free of visible dust/debris before leaving the contained work area. 15. Worker clothing must be clean and free of visible dust before leaving the work area anteroom. 16. Workers must wear shoe covers prior to entry into the work area. Shoe covers must be changed prior to exiting the anteroom to the occupied space (non-work area). Damaged shoe covers must be immediately changed. 17. Install an adhesive (dust collection) mat at the entrance of the contained work area based on facility policy. Adhesive mats must be changed routinely and when visibly soiled. 18. Consider collection of particulate data during work to monitor and ensure that contaminates do not enter the occupied spaces. Routine collection of particulate samples may be used to verify HEPA filtration efficiencies.

HEPA—high-efficiency particulate air filter; HVAC—heating, ventilation, and air conditioning.

## Data Availability

Data sharing not applicable. No new data were created or analyzed in this study. Data sharing is not applicable to this article.
